# Advancing chromosomal-scale, haplotype-resolved genome assembly: beading with Hi-C data

**DOI:** 10.1007/s44307-024-00035-7

**Published:** 2024-09-03

**Authors:** Kesen Zhu, Qingyun Li, Qianqian Kong, Junpeng Shi

**Affiliations:** School of Agriculture and Biotechnology, Shenzhen Campus of Sun Yat-Sen University, Sun Yat-Sen University, Shenzhen, 518107 China

Assembling haplotype-resolved genome sequences is critically important not only for polyploid species but also for diploids exhibiting high heterozygosity (Kong et al. 2024). Recent research has significantly increased, focused on generating haplotype-resolved assemblies by leveraging the continuous advancements in long-read sequencing technologies (e.g., PacBio HiFi reads), three-dimensional linked reads (e.g., Hi-C and Pore-C), and sophisticated assembly algorithms. Despite these advancements, several challenges persist in haplotypic assemblies, including the necessity of parental information when employing the Trio-binning method, the frequent occurrence of mis-joins between allelic chromosomes, and the reliance on a chromosomal-scale reference genome for haplotype scaffolding. In response to these challenges, Zeng and his colleagues reported an innovative algorithm named HapHiC (Zeng et al. 2024), demonstrating its superior performance in achieving chromosomal-scale, haplotype-resolved genome scaffolding without the requirement of a reference genome.

## Progress of haplotype-resolved genome assemblies

**Fig. 1:**
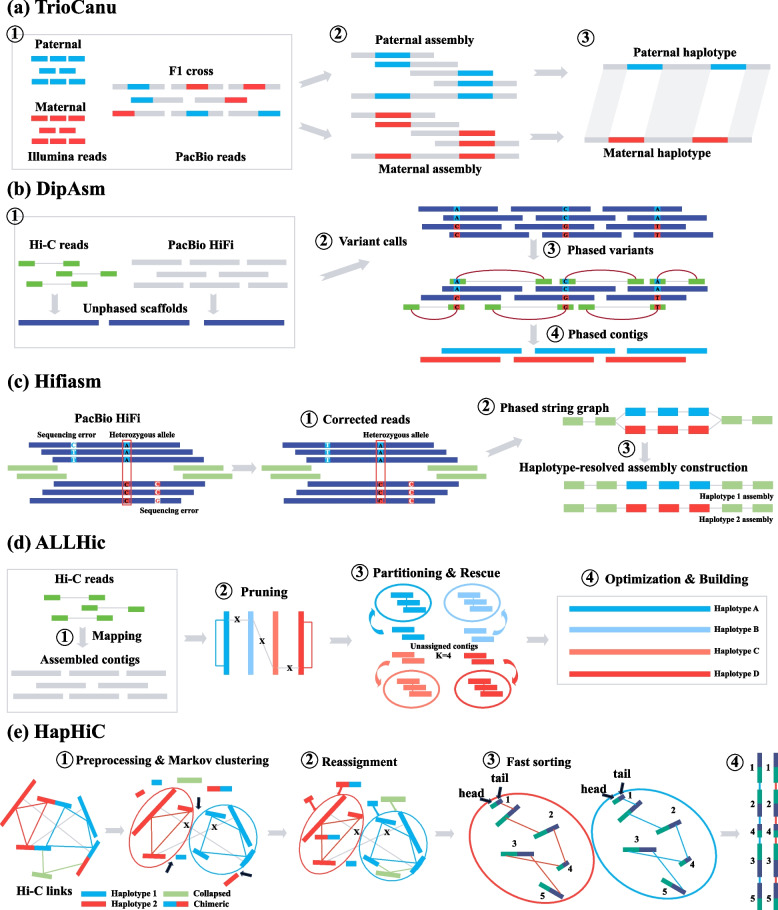
Haplotype-resolved assembly pipelines of TrioCanu, DipAsm, Hifiasm, ALLHiC and HapHiC. **a** TrioCanu: This pipeline utilizes reads from two parents to assign and independently assemble reads in the offspring. Paternal and maternal reads are depicted in blue and red, respectively. **b** DipAsm: An overview illustrates the phased assembly algorithm. Dark blue bars represent reads containing heterozygous alleles, which are progressively clustered to generate locally phased contigs. **c** Hifiasm: Similar to DipAsm, Hifiasm offers a more advanced approach and includes three distinct modes: HiFi-only assembly mode (as shown in the figure), which uses only HiFi data; Trio-Binning mode, which combines HiFi data with parental short-reads data; Hi-C integrated assembly mode, which integrates HiFi data with Hi-C data. Blue bars represent reads with heterozygous alleles carrying local phasing information, while green bars indicate reads from homozygous regions without heterozygous alleles. **d** ALLHiC: Inputs include assembled contigs, Hi-C reads, and a reference genome. Haplotype-specific chromosomes and contigs are shown in distinct colors, representing the separation of haplotypes. **e** HapHiC: A schematic graph illustrating contigs connected by Hi-C links. Contigs from haplotypes 1 and 2 are represented by red and blue rectangles, respectively. Collapsed and chimeric contigs are depicted as green and bicolored rectangles. Inter-allelic Hi-C links are indicated by grey lines. Before chromosome assignment, each contig and its associated Hi-C link undergo a preprocessing step. During this step, Hi-C links between allelic contig pairs are removed (indicated by black crosses), and breakpoints for assembly correction are marked by black arrows. Unfavorable Hi-C signals and misassembled contigs are temporarily removed before Markov clustering. The filtered contigs are then reassigned to the most appropriate cluster, and each contig is rapidly sorted using an accelerated 3D-DNA iterative scaffolding algorithm

Haplotype-resolved assembly in diploid organisms involves the separation and subsequent assembly of sequencing reads from two distinct haplotypes. In contrast, assembling haplotypes in polyploid organisms presents greater challenges due to the increased number of chromosome combinations and interactions. ‌To address these technical complexities, several advanced tools have been developed, including TrioCanu (a module of the Canu assembler), Dipasm, Hifiasm, and ALLHiC (Fig. 1a-d). TrioCanu performs haplotype-resolved assembly by utilizing the similarity between long reads of offspring and short reads from two parents (Koren et al. 2018). However, some reads remain ambiguously classified, leading to repeated or incorrect assemblies. Dipasm and Hifiasm capitalize on PacBio HiFi long reads combined with Hi-C data to efficiently generate chromosome-level haplotype-resolved genomes (Cheng et al. 2021; Garg et al. 2021). Hifiasm demonstrates superior capabilities compared to Dipasm, largely due to its integrated graph-binning strategy, which effectively resolves haplotype phases in complex genomic regions. In scenarios where only PacBio HiFi long reads are available, Hifiasm can directly phase the raw assemblies into primary and alternate contigs, while chromosomal-scale phased genomes can be generated when both HiFi reads and Hi-C data are available. Despite extensive testing and optimization in diploids, these methods have been less frequently applied to haplotype assemblies in polyploids. ALLHiC, a Hi-C scaffolding tool specifically designed for haplotype phasing in autopolyploids, requires a chromosome-level reference genome (Zhang et al. 2019). It has been successfully applied to autopolyploid sugarcane, autotetraploid alfalfa, and diploid genomes (Chen et al. 2020; Zhang et al. 2018, 2021). All these assemblers exhibit unique strengths that contribute to the effective construction of haplotype-resolved genomes.

The use of reference genomes (e.g., ALLHiC) or parental reads (e.g., TrioCanu) enhances the accuracy of haplotypic assembly and scaffolding but also introduces considerable biases and limitations. For example, reference genomes may fail to accurately capture variations in chromosome synteny within a species, particularly in polyploids characterized by high heterozygosity and extensive structural variations. Regarding the Trio method, the dependence on parental data imposes additional constraints on its practical application, particularly in hybrid plants or species with unknown or inaccessible parental information (Kong et al. 2024). Hifiasm utilizes Hi-C sequencing data for chromosome-level phasing without the need for parental data. Although Hifiasm typically achieves high accuracy in diploid genome assembly, it struggles with imbalanced phasing results when applied to autopolyploid genomes. ALLHiC excels in haplotype phasing for polyploids, despite its reliance on a pre-assembled, chromosome-level reference genome from a closely related species. Consequently, despite significant advancements in assembling haplotype-resolved genomes, these methods continue to face challenges related to the prerequisite of specific data resources, especially for autopolyploids.

## HapHiC: an innovative method in haplotype scaffolding

Scaffolding contigs generated by haplotype-resolved assemblers is a crucial step in constructing chromosomal-level haplotype-resolved genomes. The major challenges in haplotype scaffolding include strong Hi-C signals between allelic contigs, erroneous merging of contigs from different haplotypes, and chimeric contigs arising from false connections (Guk et al. 2022; Yuan et al. 2021). Therefore, an effective scaffolding method must accurately identify allelic-aware haplotypes and demonstrate robust tolerance to various assembly errors. HapHiC, a recently introduced tool, addresses these challenges by progressively breaking chimeric contigs, clustering contigs, removing allelic links, and exhibiting better tolerance to assembly errors (Zeng et al. 2024). Its innovative algorithms precisely discern patterns in Hi-C signals associated with assembly errors and allelic contig pairs. By harnessing Hi-C data, HapHiC efficiently assigns chromosomes, rapidly orders contigs, and determines their orientation, without relying on a reference genome, unlike ALLHiC (Fig. 1e). Compared to other Hi-C tools (LACHESIS, 3D-DNA, SALSA2 and YaHS), HapHiC has shown versatility in handling both simulated and truthful data across diverse taxa and ploidy levels. Given its advantages, HapHiC is poised to play a significant role in advancing chromosomal-scale, haplotype-resolved genomes, especially in non-model and autopolyploid organisms.

However, HapHiC presents a set of its challenges. Achieving high-quality haplotypic contigs remains a fundamental requirement for effective haplotype construction. Despite employing advanced algorithms (e.g., rank-sum method), HapHiC struggles to adequately address collapsed and chimeric contigs, particularly in cases of low sequence divergence. Another significant challenge is the precise clustering of contigs, which often requires prior knowledge of chromosome numbers. HapHiC attempts to address these problems by manually establishing chromosomal boundaries through rapid sorting without clustering, but this approach introduces potential bias. Additionally, while HapHiC permits manual adjustment of chromosome boundaries, this feature requires a certain level of expertise. The inherent difficulties in resolving collapsed regions and accurately clustering contigs highlight the ongoing need for refining allele-aware scaffolding methodologies, which is crucial for fully realizing the potential of HapHiC and similar tools.

## Perspectives of haplotype-resolved genome assembly

Haplotype-resolved genome assembly represents a significant advance in decoding the genomic diversity not only among individuals but also between homologous chromosomes within a single individual. Haplotype-resolved genomes can greatly enhance downstream genome analyses, such as generating a bi-parental genome graph to facilitate allele-specific gene expression profiling (Kong et al. 2024). Concurrently, the development of telomere-to-telomere (T2T) genome assemblies has marked a significant leap forward, establishing a new frontier in genome assembly (Li and Durbin 2024). In this rapidly advancing field, achieving haplotype-resolved assembly at the T2T chromosomal level has become a critical research objective. Despite these prospects, haplotypic genome assembly currently faces several limitations, particularly challenges in accurate contig clustering. To fully leverage the capabilities of tools like HapHiC, it is essential to establish new standards for aligning, filtering, and analyzing Hi-C data. Emerging technologies, such as Pore-C, which captures higher-order chromatin conformations through multi-way contacts (Deshpande et al., 2022), have the potential to resolve low-heterozygosity regions between haplotypes. Furthermore, integrating additional data types, such as ultra-long sequencing reads from Nanopore technologies, could enhance the accuracy and completeness of haplotype-resolved assemblies, driving future advancements in decoding haplotype variations.
